# Influence of parboiling conditions on rice grain quality characters and insect infestation with rice weevil (*Sitophilus oryzae*. L) of some rice cultivars

**DOI:** 10.1186/s12870-024-05651-y

**Published:** 2024-10-17

**Authors:** Khaled M. H. Abd El Salam, Germine M. Abou El-Soud, Abd El Salam M. Marei, Khaled H. M. Abdel-Rheim, Ahmed Abdel‑Megeed, Sobhi F. Lamlom

**Affiliations:** 1 Rice Technology Training Center (RTTC), Field Crops Research Institute, Agricultural Research Center, Alexandria, Egypt; 2 Cereals and Stored Product Insects Research Department, Plant Protection Research Institute, Agricultural Research Center, Alexandria, Egypt; 3 Department of Plant Protection, Faculty of Agriculture, (Saba‑Basha), Alexandria University, Alexandria, Egypt; 4 Plant Production Department, Faculty of Agriculture Saba Basha, Alexandria University, Alexandria, 21531 Egypt

**Keywords:** Parboiling, Soaking, Grain quality characters, *Sitophilus oryzae*, Index of susceptibility

## Abstract

Parboiling improves rice grain hardness and reduces susceptibility to *Sitophilus oryzae* infestation by gelatinizing the starch and enhancing resistance.A newly designed electric machine was used to parboil four Egyptian rice cultivars—Sakha 108, Giza 178, Super 300, and Egyptian Yasmin—at 70, 75, and 80 °C and determine their susceptibility to *Sitophilus oryzae* L. (Coleoptera: Curculionidae) infestation. Results indicated that heating affected most traits in all four rice cultivars during both study seasons 2021 and 2022. Super 300 rice cultivar exhibited the highest hulling values (81.23 and 81.42%) when heated to 80 °C, while the Yasmin rice cultivar showed the lowest values for hulling (77.66 and 77.45%) at 70 °C. while Giza 178 cultivar showed a significant decrease in broken percentage (90.85 and 94.02%) compared to control when heated to 80 °C. The results also indicated that the Yasmin rice cultivar had the highest values for white belly, hardness, and gel consistency at 80 °C, while the Sakha 108 cultivar showed the lowest values for these traits at 70 °C. Furthermore, the protein, elongation, and water uptake characters significantly responded to the different investigated treatments. Yasmin cultivar at 80 °C showed the highest significant values for protein (9.26 and 9.47%), elongation (65.02 and 65.44%), and water uptake (453.2 and 455.1 ml water/100 g milled grains) in both seasons. Sakha 108 cultivar had the lowest values for these traits at 70 °C. The S. oryzae insects responded differently to the rice cultivars. Using Dobie’s Index of Susceptibility, all cultivars were classified as resistant to S. oryzae infestation. Super 300 was moderately resistant before parboiling but resistant after heat treatment. In conclusion, the study underscores the influence of pre-storage parboiling on rice weevil infestation, suggesting that heat treatment could serve as an effective control measure. These findings emphasize the importance of parboiling conditions in enhancing rice grain quality and bolstering resistance to insect infestation.

## Introduction

Rice, scientifically known as *Oryza sativa* L., is cultivated in more than 100 nations and regions. It serves as a crucial source of energy for over half of the global population [1, 2]. The primary technique for obtaining rice-based foods is the conversion of rice into powder. Rice-based delicacies, such as rice bread, rice cakes, sweet dumplings, and rice noodles, are influenced by the type of rice and the way it is processed, which have a substantial impact on the overall quality of the finished product [3]. Various types of rice display differences in texture, color, and nutrient composition [4, 5]. The milling process has an impact on various characteristics and physicochemical features of rice flour, such as the amount of starch, the size and distribution of particles, the process of gelatinization, and the thermal properties [6–10]. These characteristics subsequently impact the overall quality of the product.

Parboiling, an age-old practice found in parts of Asia, is particularly prevalent in India, Africa, and to a lesser extent in some European countries and the United States, is a hydrothermal treatment applied to paddy rice [11]. This treatment enhances the storage stability of rice and involves a sequence of steps including soaking or steeping, steaming, and drying [12]. Its benefits include improving the milling recovery of paddy rice, salvaging poor-quality spoiled paddy rice, and meeting specific consumer demands. The process of parboiling rice maintains the nutrients of the outer branny layers, which are typically lost during the polishing of milled rice (MR). It also leads to an increase in head rice yield (HRY), enhanced phytochemical and biochemical characteristics, higher concentration of water-soluble vitamins, denaturation of the bran layers by heat, lipase activity and oxidative rancidity, increased stickiness, and changes in cooking properties, textural properties, and sensory attributes [13]. Thus, parboiling of paddy is crucial for enhancing storage longevity, milling conditions, and cooking characteristics. The cooked parboiled rice exhibits superior firmness, stickiness, and higher vitamin B1 content in comparison to the MR. In their study, Sumczynski, Bubelova, and Fisera [14] showed that parboiled rice has superior digestibility, so indicating its positive impact on human health. Rice has notably elevated concentrations of specific phenolic acids, including ferulic, p-coumaric, and caffeic acids. Research findings indicated that the storage properties of free form (FF) and bound form (BF) phenolic and flavonoid concentrations, as well as the corresponding antioxidant capacities, in red and purple bran rice were notably superior to those of light-colored bran rice and other cereals [15]. Alongside traditional methods, various modern techniques have been developed worldwide, utilizing either new or existing technologies to enhance product quality [16, 17]. The extent of parboiling, especially concerning starch gelatinization, significantly influences attributes such as milling quality and physicochemical characteristics of parboiled rice [18, 19]. While soaking rice at room temperature is widespread, it requires a considerable duration to reach a moisture content of approximately 30 g/100 g wb. Warm- or hot-water soaking is often employed to shorten this duration, as higher temperatures accelerate hydration rates [20, 21]. However, it’s recommended to maintain soaking temperatures below the point of starch gelatinization to minimize kernel splitting and the subsequent loss of solids and phytochemicals [22]. Many parboiled rice plants choose traditional methods to prevent excessive solid leaching during warm water soaking. These techniques usually entail immersing in cold water and subjecting to steam at a temperature of 100 °C [12]. Nonetheless, a challenge arises when cracks develop in the final product, possibly due to the steam treatment in large cylindrical tanks, leading to rice splitting at the bottom [21]. Additionally, excessive water absorption during parboiling can contribute to rice kernel splitting [23]. Thus, reducing soaking time is essential to prevent this issue, as well as utilizing appropriate shelving to minimize pressure on the rice during steaming. Tumbling involves rolling paddy rice and water together inside a tumbler for varying durations to facilitate water absorption into the rice kernels, replacing traditional soaking [24]. Tempering entails allowing wet paddy rice to rest at room temperature for specified durations to equalize moisture content within the rice kernels. Magnetic resonance imaging has objectively demonstrated a decrease in moisture gradient within rice kernels during tempering [25]. In the parboiling process, soaking is a crucial phase that allows the diffusion-controlled water absorption to move into the rice kernel where it changes the gross composition and distribution of nutrients inside the grain [26], which affects subsequent processing activities such as storage, milling, cooking, and eating quality [27–30]. The initial soaking temperature and time are critical parameters in rice parboiling and have a considerable impact on the quality of milled parboiled rice [31]. Improper soaking at low temperatures results in microbial contamination, whereas soaking at high temperatures results in surface sloughing before optimal hydration [32]. Leaching loss, fermentation, kernel rupture, and color change occur as a result of prolonged soaking [12, 33–35]. Similarly, Mir and Bosco investigated the impact of various soaking temperatures on the functional and physical characteristics of rice cultivars [36]. The hardness value increases as the soaking temperature rises, which will aid in improving rice’s yield during milling. In addition, it has been observed that the water soaking temperature of 40 °C, 50 °C, and 60 °C affects the physicochemical, milling, and cooking characteristics of parboiled rice [26, 37].

The quality of food grains is significantly impacted by the level of insect infestation [38]. Due to their ability to infest paddy, milled rice, and mill by-products, insects have the potential to induce significant economic losses. One of the most common insect pests affecting stored food grains has been identified as the rice weevil (*Sitophilus oryzae*) [39].Female weevils lay their eggs inside the rice kernel, where the larvae mature into adult weevils, and the adults consume the rice. While the thermal step of parboiling can be used to sterilize rice against field pests, parboiling rice is also a cost-effective technique to increase storage stability (reducing insect infestation). This is due to changes in the mechanical properties of the rice grain, making it less penetrable by weevils. Parboiled rice was quite resistant to attack by many externally feeding insects [40]. The rice weevil (*Sitophilus oryzae*) is one of the most dangerous pests of whole grains. Heavy infestations can cause spoilage and decomposition of stored products. The flour beetles doesn’t just cause damage by feeding, but also cause further problems by contaminating the stored products, with large numbers of dead bodies, cast skins, and fecal pellets, as well as liquids secretions, and pungent odours in grain [41]. In addition, losses due to weevils alone can exceed 25% of the crop and may even reach 40% [42]. The development of these weevils also affects the physico-chemical properties of the resulting flours. Gabarty and Abou El Nour [43], showed a significant increase in the total protein contents and a decrease in monosaccharides and disaccharides in sieved and residual wheat flour samples infested with *Corcyra cephalonica, Ephestia kuehniella, and Tribolium confusum*. Furthermore, heat sterilization and parboiling of rice may be crucial in improving the storage quality of rice since weevil populations often develop in non-sterilized grains while a low degree of resistance appears in non-sterilized grains [44]. This study aims to determine the appropriate soaking temperature for four Egyptian rice cultivars, namely Sakha 108, Giza 178, Super 300, and Egyptian Yasmin, to obtain parboiled rice using a new technique based on a newly designed local electric machine and evaluate their susceptibility to infestation with *S. oryzae*.

## Materials and methods

### Study site and rice cultivars

The experiment was conducted at the Rice Technology Training Center (RTTC) in Alexandria, Egypt. Four rice cultivars were used to assess the effects of temperature on grain quality characteristics. The rice cultivars tested included Giza 178 (indica/japonica), Egyptian Yasmin (indica), Sakha 108 (japonica), and Super 300 (a hybrid rice variety). Newly harvested, certified seeds from the 2021 and 2022 growing seasons were provided by the Rice Research Program, Agricultural Research Center, Sakha, Kafr El-Sheikh, Egypt.

Additionally, the susceptibility of these rice cultivars—both in paddy and milled forms, including parboiled and un-parboiled rice—to the rice weevil (*Sitophilus oryzae*) was evaluated. These susceptibility studies were conducted at the Cereals and Stored Product Insects Research Department, Plant Protection Research Institute, Agricultural Research Center, Sabahiya, Alexandria, Egypt.

### Parboiling process

**Fig. 1:**
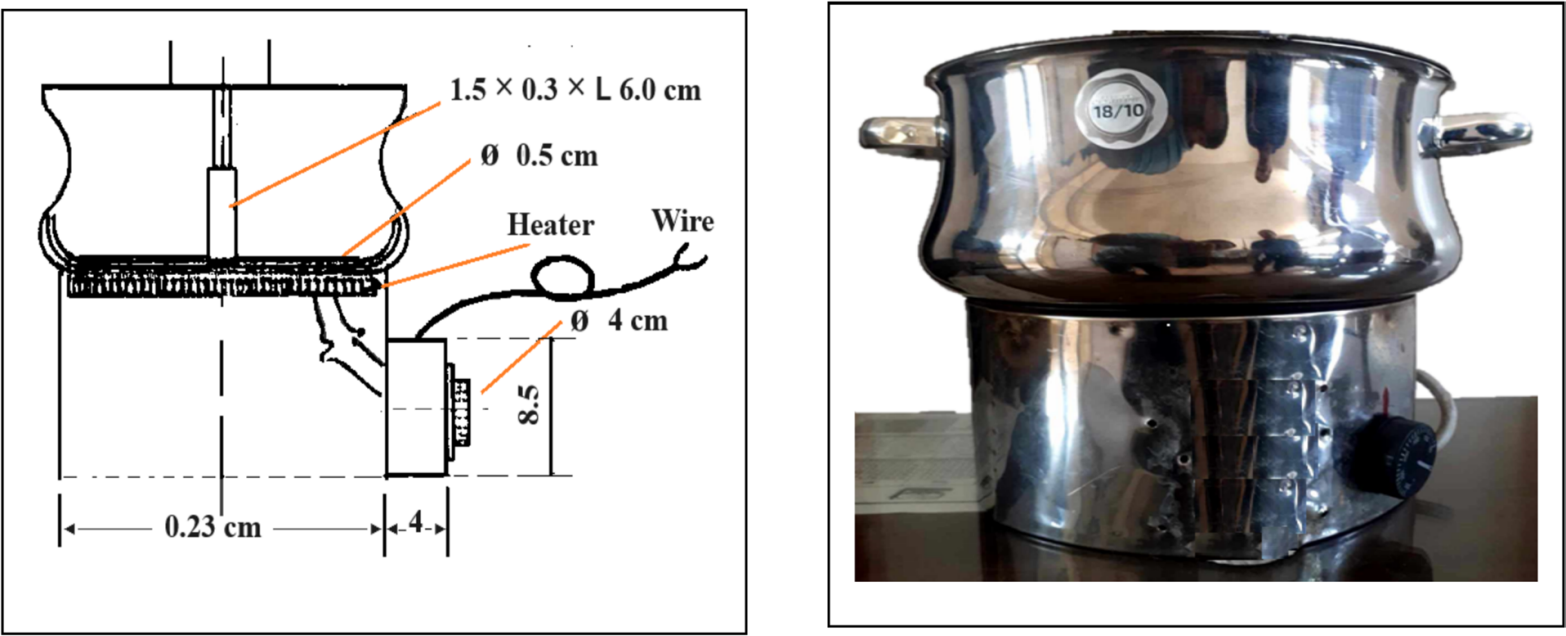
Schematic drawing of electric parboiling machine

Newly harvested paddy rice grains from four rice cultivars (1 kg of paddy rice for each cultivar) were soaked for 2.5 h in jute sacks at 70 °C, 75 °C, and 80 °C. Following the soaking, the rice grains were subjected to brief steaming for 5–8 min at a temperature slightly below boiling (~ 98 °C), using a locally designed electrical parboiling machine. The parboiling process was intended to gelatinize the starch without fully cooking the rice grains. The equipment consisted of a stainless-steel vessel (23 cm internal diameter, 8 cm top cover, and 603.2 cm^3^ internal volume), with temperature monitored by a thermocouple linked to a voltage regulator to ensure consistency throughout the process.. Figure 1 was designed and manufactured to study different parboiling conditions. The equipment consists of a stainless-steel vessel with physical dimensions of 23 cm internal diameter, 8 cm top cover, and 603.2 cm^3^ internal volume. A thermocouple linked to a voltage regulator measures the temperature inside the parboiling pot.

### Studied characters

Randomly selected rice samples (each weighing 150 g) were cleaned by a Dockage analyzer machine (Carter Day CO, USA) to automatically remove foreign matter, immature green balls, and clay balls, processed in a Satake polishing mill, and experimentally dried with a Satake huller machine, evaluated according to IRRI [45]. Amylose content was determined according to improved techniques [46]. Gel consistency was evaluated [47], and the gelatinization temperature was measured [48]. Brown rice protein content was calculated using the standard Micro-Kjeldahl technique [49]. To calculate the estimated protein content, the specific nitrogen content was multiplied by a factor of 5.95. Water uptake of milled rice samples was measured at 77 °C; according to USDA, (1965). The elongation percentage was determined according [50]. Vitamins were estimated according [51].The data were deposited in Table 1.

**Table 1:** Characteristics of milling, cooking, and eating quality for cultivars throughout the 2021 and 2022 growing seasons. (Non-parboiled rice)

Characters	Sakha 108		Giza 178		Super 300		Egyptian Yasmin	
	**2021**	**2022**	**2021**	**2022**	**2021**	**2022**	**2021**	**2022**
**Hulling %**	78.65	78.96	79.20	79.72	80.15	80.37	77.11	77.28
**Milling %**	70.56	70.83	69.45	69.76	72.30	72.14	68.20	68.42
**Broken %**	6.23	7.51	8.20	7.36	6.59	7.25	9.25	9.03
**White belly**	2.85	2.77	3.56	3.20	2.81	2.63	3.10	3.36
**Hardness (Kg/cm**^**2**^**)**	5.22	5.60	6.26	5.90	5.73	5.89	7.22	8.10
**Gel Consistency (mm)**	86.10	87.56	89.22	90.82	85.30	87.15	85.63	86.11
**Protein %**	7.13	7.37	7.73	8.26	7.12	7.33	8.25	8.53
**Elongation %**	53.31	55.10	59.41	59.92	56.13	55.8	60.42	61.21
**Water uptake (ml /100 g)**	421.3	423.6	435.1	438.2	431.6	433.4	446.3	449.5
**Vitamin B1 (mg/kg)**	1.38	1.40	1.61	1.63	1.48	1.53	1.62	1.65
**Vitamin B2 (mg/kg)**	0.13	0.14	0.16	0.17	0.14	0.15	0.18	0.18
**Vitamin B3 (mg/kg)**	45.10	45.82	50.21	50.45	47.50	48.12	53.20	54.65
**Vitamin B5 (mg/kg)**	8.35	8.38	8.96	9.22	8.62	8.66	9.93	10.13
**Vitamin B6 (mg/kg)**	4.21	4.28	4.73	4.76	4.33	4.37	5.10	5.32

### Insect culture

*S. oryzae* adults were reared in glass jars (250 mL) containing approximately 50 g of rice. Each jar was covered with a muslin cloth and secured with rubber bands for egg lying to obtain the large numbers of adults needed for the tests and incubated at 30 ± 2 °C and 65 ± 5% relative humidity.

### Susceptibility studies

Resistance levels in rice cultivars to *S. oryzae* infestations have been determined in laboratory experiments. Fifty grams of rice (paddy, milled parboiled rice, and paddy, milled un-parboiled) were weighed accurately and stored in a 250 ml glass jar. Twenty newly emerged adults from the insect species tested were introduced into glass jars covered with muslin cloth and tied with rubber bands. Adults were left for 14 days and then removed; the jars were preserved under experimental conditions. The emerging F_1_ populations in each glass jar were removed after counting every 2 days so that re-emergence did not occur, and it was expected that all F_1_ progenies would emerge before the F_2_ generation began (Bashir, 2002). The susceptibility index (SI) was calculated using the method of Dubie and Kilminster (1977), as follows:

$$\mathbf{I}\mathbf{S}=100\times \frac{\text{Natural log F}1}{\text{MDT}}$$

where DIS = Dobie Susceptibility Index, F_1_ = total number of first-generation emerging adults, and MDT = the median developmental period in days. The Dobie susceptibility index, which ranges from 0 to 11, was used to classify rice cultivars into susceptibility groups [52], where 0 to 4 is classified as resistant; 4.1 to 7.0 is classified as medium resistance; 7.1 to 10.0 were classified as susceptible and > 10 was classified as highly susceptible [52].

### Experimental design and data analyses

A split-plot design with three replicates was employed, where the main plot factor was the rice cultivars, and the sub-plot factor was the parboiling temperature. Data were analyzed using two-way ANOVA to assess the effects of cultivars, temperature, and their interaction on various traits. Fisher’s Least Significant Difference (LSD) test was used for post-hoc comparisons, with significance determined at a 5% level (*p* < 0.05). The Dobie Susceptibility Index (SI) was calculated for evaluating pest resistance, according to the following function.

$$\mathrm{IS}=100\times(\ln(\mathrm F1))/\mathrm{MDT}$$

where F1 represents the total number of first-generation adults emerging, and MDT denotes the median developmental time in days.

One-way ANOVA was applied to compare susceptibility among cultivars. Model assumptions were checked with the Shapiro–Wilk and Levene’s tests, and data transformations were applied as necessary. Statistical analyses were performed using SAS software, version 8 [53], and post-hoc testing was conducted with Costas, version 6.303. This rigorous framework ensures that observed differences are statistically significant and robust.

## Results

### Effect of soaking temperature on milling characteristics

Data in Table 2 show the relationship between the interaction of rice cultivars and temperature, and the means for milling characteristics. The results indicate significant differences among the applied treatments in terms of milling performance. Meanwhile, the highest hulling values (81.23 and 81.42%) were noticed with the Super 300 rice cultivar by heating up to 80 °C, whereas the minimum values for such a character (77.66 and 77.45%) were indicated with the Yasmin cultivar by heating at 70 °C in both study seasons, respectively. The Super 300 cultivar showed prevalence values for milling (72.95 and 73.12%) by heating up to 80 °C. Nonetheless, the lowest values for such character (68.42 and 68.56%) were declared by heating the Yasmin cultivar to 70 °C in seasons 2021 and 2022, respectively. Also, the data as shown in Table 2 declared that hulling % increased by 1.34, 1.30%, and milling % increased by 0.89, and 1.35%, respectively, for both seasons than control, because of higher cargo percentage (brown rice) and more milled rice. Furthermore, data in Table 2 indicated that the Yasmin rice cultivar gave the highest values for broken (2.21 and 2.52%) by heating at 70 °C; however, the reduced values for such character (0.75 and 0.44%) were noticed with the Giza 178 cultivar by heating up to 80 °C in both seasons, respectively. These results indicate that in Giza 178 cultivar broken percent decreased by 90.85 and 94.02% than control in both study seasons, respectively.

**Table 2:** Mean values for milling characters as affected by an interaction between cultivars and soaking temperature in the 2021 and 2022 seasons

**Cultivars**	**Soaking temperature** ^**o**^**C**	**Hulling** %		**Milling** %		**Broken** %	
		**2021**	**2022**	**2021**	**2022**	**2021**	**2022**
**Sakha 108**	70	79.13	79.33	71.32	71.51	1.55	1.63
	75	79.28	79.51	71.60	71.75	1.41	1.25
	80	79.46	79.68	71.76	71.89	1.28	1.16
**Giza 178**	70	79.68	79.89	70.28	70.49	1.21	1.05
	75	79.97	80.23	70.55	70.76	0.88	0.61
	80	80.29	80.57	70.73	70.87	0.75	0.44
**Super 300**	70	80.55	80.74	72.60	72.49	1.69	1.44
	75	80.97	81.22	72.82	72.96	1.31	1.13
	80	81.23	81.42	72.95	73.12	1.22	1.03
**Yasmin**	70	77.66	77.45	68.42	68.56	2.21	2.52
	75	77.95	77.82	68.61	68.75	1.73	2.21
	80	78.20	77.98	68.80	68.96	1.42	1.66
**L.S.D **_**0.05**_		0.002	0.051	0.034	0.025	0.012	0.017

### Effect of soaking temperature on white belly, hardness, and gel consistency characters

Regarding the response of the white belly, hardness, and gel consistency characters as impacted by the interaction between cultivars and temperature, the data presented in Table 3 make it clear that there were significant differences among the investigated treatments about the white belly, hardness, and gel consistency characters. Furthermore, the superior values for the white belly (0.47 and 0.58) were noticed with the Giza 178 cultivar at 70 °C, while the Sakha 108 cultivar demonstrated the lowest results for such a trait (0.13 and 0.17, respectively) by heating to 80 °C in both seasons. The results indicated a decrease in white belly, especially in the Sakha 108 cultivar, by 95.43 and 93.86% compared to control in both study seasons, respectively.

**Table 3:** Average values of white belly, hardness, and gel consistency as affected by the interaction between cultivars and temperature in the 2021 and 2022 seasons

**Cultivars**	**Soaking Temperature** (^o^C)	**White belly**		**Hardness** (Kg/cm^2^)		**Gel consistency** (mm)	
		**2021**	**2022**	**2021**	**2022**	**2021**	**2022**
**Sakha 108**	70	0.30	0.46	10.70	10.96	88.40	88.67
	75	0.23	0.35	10.95	11.21	89.85	90.12
	80	0.13	0.17	11.22	11.39	90.67	91.22
**Giza 178**	70	0.47	0.58	10.38	10.66	90.64	91.32
	75	0.31	0.42	10.61	10.82	92.09	92.81
	80	0.20	0.32	10.85	11.18	92.90	93.59
**Super 300**	70	0.34	0.55	9.62	9.87	88.71	89.03
	75	0.25	0.40	10.37	10.62	90.44	90.75
	80	0.20	0.26	10.49	10.85	90.68	90.91
**Yasmin**	70	0.38	0.53	12.15	12.36	92.39	93.60
	75	0.23	0.37	12.44	12.60	94.56	95.70
	80	0.17	0.23	12.77	13.12	95.48	96.39
**L.S.D **_**0.05**_		0.013	0.029	0.219	0.207	0.134	0.211

Furthermore, results in Table 3 showed that the greatest values for gel consistency (95.48 and 96.39 mm) were seen with the Yasmin cultivar at 80 °C, while the lowest values (88.40 and 88.67 mm) were observed with the Sakha 108 cultivar at 70 °C in both seasons. Additionally, results in Table 3 showed that the Yasmin cultivar showed the highest values for hardness at 80 °C (12.77 and 13.12 kg/cm^2^), but the minimum values for such character (9.62 and 9.87 kg/cm^2^) were indicated with the Super 300 cultivar at 70 °C in both study seasons, respectively. Hardness increased in the Yasmin cultivar by 76.86 and 61.97% and gel consistency by 11.50 and 11.93% compared to the control in both seasons, respectively.

### Effect of soaking temperature on protein, elongation, and water uptake characters

The findings in Table 4 revealed significant responses of protein, elongation, and water uptake characteristics to various treatments involving the interaction of cultivars and temperature. The highest significant values for protein (9.26 and 9.47%) were indicated with the Yasmin rice cultivar by heating up to 80 °C, while the Sakha 108 cultivar was found to have the lowest values for this trait (8.11 and 8.26%) at 70 °C in both seasons. Additionally, the Yasmin cultivar at 80 °C treatment gave the maximum values for elongation (65.02 and 65.44%). The minimum values for such character (56.90 and 57.32%) were indicated with the Sakha108 cultivar by heating up to 70 °C in both seasons, respectively. Yasmin cultivar at 80 °C showed the highest significant values 453.2 and 455.1 ml water/100 g milled grains in both seasons respectively. In contrast, the Sakha108 cultivar had the lowest values for this characteristic at 70 °C in both seasons, at 425.1 and 426.6 ml water/100 g milled grains, respectively. Yasmin cultivar showed a superior increase in protein by 12.24 and 11.01%, elongation of rice kernels by 7.61 and 6.91%, and water uptake by 1.54 and 1.24% compared to control in both seasons, respectively.

**Table 4:** Average values of protein, elongation, and water uptake characters affected by the interaction between cultivars and temperature in the 2021 and 2022 seasons

**Cultivars**	**Soaking Temperature** (^o^C)	**Protein** %		**Elongation** *%*		**Water uptake** (ml/100 g)	
		**2021**	**2022**	**2021**	**2022**	**2021**	**2022**
**Sakha 108**	70	8.11	8.26	56.90	57.32	425.1	426.6
	75	8.38	8.52	58.11	58.57	428.3	429.5
	80	8.57	8.75	58.64	58.92	430.8	431.3
**Giza 178**	70	8.56	8.71	61.08	61.41	442.3	444.2
	75	8.82	9.22	62.12	62.50	443.1	445.3
	80	9.12	9.39	61.96	62.83	445.3	446.9
**Super 300**	70	8.25	8.52	57.87	58.33	433.7	435.3
	75	8.68	8.81	59.09	59.56	436.1	437.6
	80	8.83	9.03	59.45	59.80	437.2	438.3
**Yasmin**	70	8.83	9.02	63.86	64.33	449.3	452.2
	75	9.11	9.30	64.40	64.92	451.5	453.8
	80	9.26	9.47	65.02	65.44	453.2	455.1
**L.S.D **_**0.05**_		0.022	0.214	0.251	0.141	0.522	0.861

### Effect of soaking temperature on B group vitamins

The data presented in Table 5 indicate that the interaction between rice cultivars and temperature significantly affected the vitamin content of the studied rice cultivars. The Yasmin cultivar heated to 80 °C had the highest values for vitamin B1 (1.98 and 2.12 mg/kg rice), vitamin B2 (0.197 and 0.206 mg/kg rice), vitamin B3 (58.75 and 59.33 mg/kg rice), vitamin B5 (11.33 and 11.58 mg/kg rice), and vitamin B6 (5.81 and 5.92 mg/kg rice) in both the 2021 and 2022 seasons, respectively. In contrast, the Sakha108 rice cultivar heated to 70 °C had the lowest values for vitamin B1 (1.44 and 1.49 mg/kg rice), vitamin B2 (0.141 and 0.145 mg/kg rice), vitamin B3 (47.49 and 48.94 mg/kg rice), vitamin B5 (8.53 and 8.71 mg/kg rice), and vitamin B6 (4.58 and 4.71 mg/kg rice) in the 2021 and 2022 seasons, respectively. Compared to the control, the Yasmin rice cultivar showed superior increases in vitamin B1 by 22.22% and 28.48%, vitamin B2 by 12.57% and 15.73%, vitamin B3 by 10.43% and 8.56%, vitamin B5 by 14.09% and 14.31%, and vitamin B6 by 13.92% and 11.27% in both study seasons, respectively.

**Table 5:** Average values for the characters of vitamin B1, vitamin B2, vitamin B3, vitamin B5, and vitamin B6, as affected by the interaction between cultivars and temperature in the 2021 and 2022 seasons

**Cultivars**	**Soaking Temperature** (^o^C)	**Vitamin B1** (mg/kg rice)		**Vitamin B2** (mg/kg rice)		**Vitamin B3** (mg/kg rice)		**Vitamin B5** (mg/kg rice)		**Vitamin B6** (mg/kg rice)	
		**2021**	**2022**	**2021**	**2022**	**2021**	**2022**	**2021**	**2022**	**2021**	**2022**
**Sakha 108**	70	1.44	1.49	0.141	0.145	47.49	48.94	8.53	8.71	4.58	4.71
	75	1.59	1.63	0.154	0.161	51.60	53.09	9.26	9.70	4.78	4.97
	80	1.64	1.67	0.162	0.168	52.56	53.64	9.55	9.94	4.90	5.24
**Giza 178**	70	1.60	1.68	0.168	0.172	50.73	51.76	9.67	10.01	4.93	4.97
	75	1.76	1.81	0.171	0.176	54.77	55.52	10.25	10.62	5.06	5.17
	80	1.79	1.83	0.180	0.183	55.35	56.25	10.47	10.91	5.18	5.41
**Super 300**	70	1.56	1.59	0.150	0.154	48.74	49.75	8.89	9.18	4.66	4.83
	75	1.62	1.69	0.162	0.166	51.40	51.66	9.53	9.88	4.94	5.10
	80	1.68	1.72	0.167	0.172	51.81	52.21	9.75	10.10	5.16	5.23
**Yasmin**	70	1.71	1.76	0.181	0.185	55.21	56.19	10.50	10.80	5.35	5.43
	75	1.80	1.85	0.192	0.195	57.49	58.26	11.09	11.35	5.54	5.68
	80	1.98	2.12	0.197	0.206	58.75	59.33	11.33	11.58	5.81	5.92
**L.S.D **_**0.05**_		0.018	0.113	0.004	0.011	0.231	0.152	0.125	0.113	0.016	0.031

### Correlation coefficient between all studied traits

**Fig. 2:**
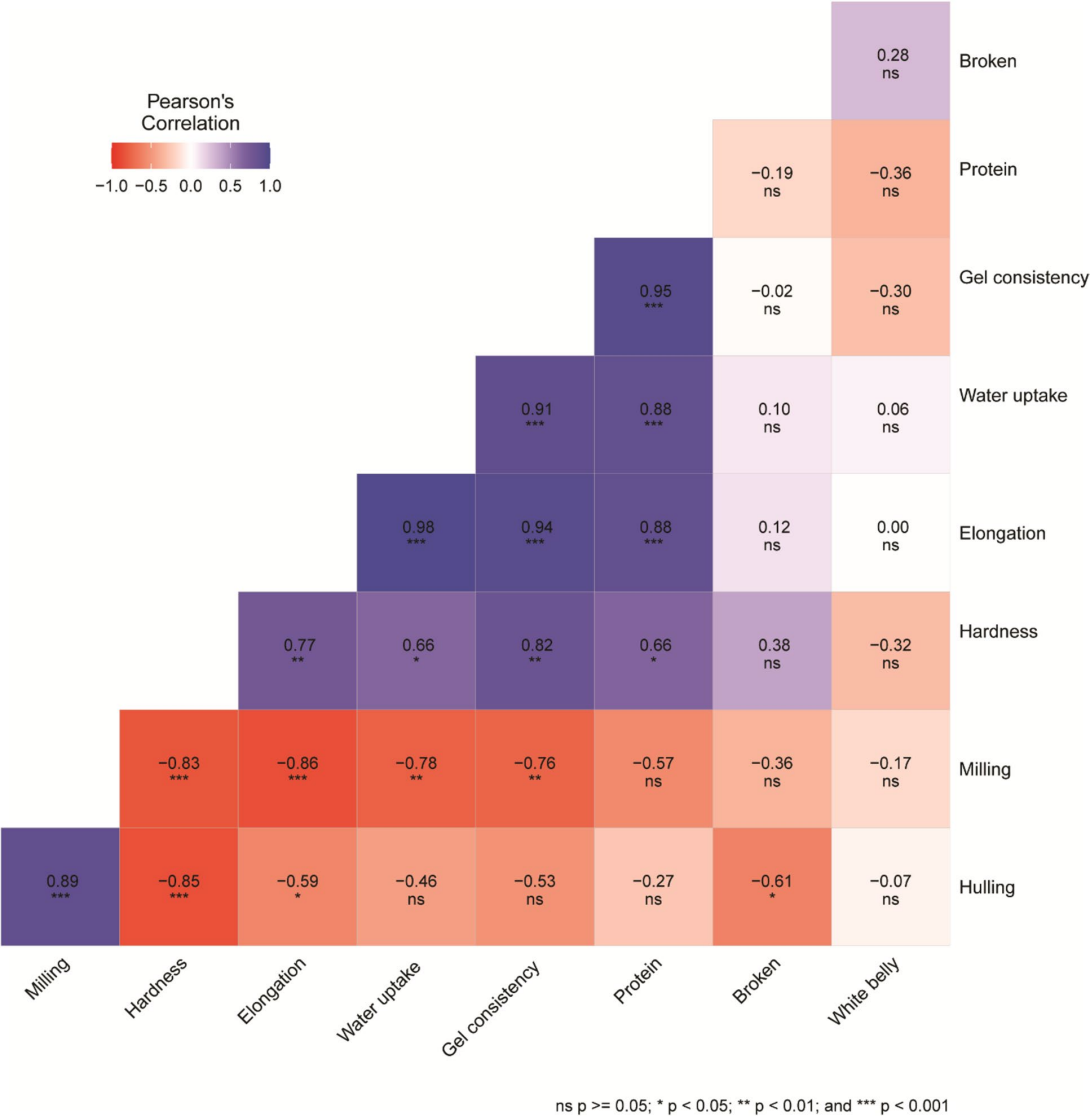
The correlation coefficient between milling, cooking, hulling, and eating quality characters in the 2021 and 2022 seasons

The data presented in Fig. 2 illustrate the relationship between milling, cooking, and eating quality traits during both seasons. The correlations observed are as follows: Hulling percentage exhibited a positive and significant correlation with milling percentage (0.89) while showing a negative correlation with elongation (0.59) and hardness (0.85). Milling percentage displayed a negative and significant correlation with hardness (0.83), gel consistency (0.76), elongation (0.86), and water uptake (0.78). Hardness demonstrated a positive and significant correlation with elongation (0.77), water uptake (0.66), gel consistency (0.82), and protein (0.66). Elongation showed a significant and positive correlation with water uptake (0.98), gel consistency (0.94), and protein (0.88). Water uptake exhibited a significant and positive correlation with protein (0.88) and gel consistency (0.91). Gel consistency displayed a positive and significant correlation with protein percentage (0.95).

### Rice grains susceptibility

#### Adult Emergence from rice cultivars

The number of *Sitophilus oryzae* adults emerging from parboiled and un-parboiled rice samples varied across Egyptian rice cultivars. Generally, fewer adults emerged from paddy rice compared to milled rice, as shown in Table 6. Among paddy-parboiled samples, the Super 300 cultivar treated at 75°C and 80°C showed the lowest emergence, with no adults emerging at the higher temperature. For milled-parboiled rice, Super 300 treated at 80°C had the fewest adults, averaging 1.67. Giza 178 and Sakha 108 paddy-parboiled samples treated at 80°C also had 1.67 adults. The Super 300 paddy and milled-parboiled samples treated at 70°C and 75°C had slightly higher emergence, with 2.50 adults. In contrast, the Yasmin cultivar had the highest number of adults in its milled-un-parboiled form (30 adults), followed by milled-parboiled Yasmin at 70°C (26.20 adults), paddy-un-parboiled Yasmin (25 adults), and milled-un-parboiled Giza 178 and Sakha 108 (20 adults). These results indicate that paddy-parboiled rice treated at 80°C is highly resistant to *S. oryzae*.

**Table 6:** Comparative classification of parboiled rice of some Egyptian rice cultivars to *Sitophilus oryzae* resistance using the Dobie index

**Cultivar**	**Condition**	**Temperature** (^o^C)	**F1 progeny emerged**		**Median development time** (days)		**S1** **(Index of susceptibility)**		**Resistance category**	
			**paddy**	**milled**	**paddy**	**milled**	**paddy**	**milled**	**paddy**	**milled**
**Sakha 108**	**Un parboiled**		18.33	20.00	58	60	2.17	2.16	R	R
	Parboiled	70	11.67	14.16	54	57	1.97	2.01	R	R
		75	9.16	9.16	55	56	1.74	1.71	R	R
		80	2.50	4.50	40	47	0.99	1.38	R	R
**Giza 178**	**Un parboiled**		15.00	20.00	61	60	1.92	2.16	R	R
	parboiled	70	10.83	16.67	61	57	1.69	2.14	R	R
		75	8.33	10.00	60	52	1.53	1.92	R	R
		80	2.50	5.00	50	45	0.79	1.55	R	R
**Super 300**	**Un parboiled**		5.00	8.33	25	19	1.83	4.84	R	M
	parboiled	70	2.50	4.50	33	29	1.20	2.25	R	R
		75	0.00	2.50	0	33	0.00	1.20	R	R
		80	0.00	1.67	0	40	0.00	0.55	R	R
**Yasmin**	**Un parboiled**		25.00	30.00	50	42	2.79	3.51	R	R
	parboiled	70	20.00	26.20	50	47	2.60	3.01	R	R
		75	15.00	21.67	51	52	2.30	2.56	R	R
		80	11.67	18.33	52	55	2.55	2.29	R	R
**L.S.D **_**0.05**_			8.61	9.38	27.8	19.9	1.00	1.28	-	-

#### The median duration of rice weevil development

The parboiling technique significantly influences the developmental stages of rice weevils across different rice cultivars, as detailed in Table 6. The study revealed that, without parboiling, Sakha 108 and Giza 178 had the longest developmental periods, taking 58 and 61 days, respectively. However, when subjected to parboiling at 80°C, their developmental times were reduced to 40 and 50 days, respectively. Conversely, Super 300 and Yasmin, which initially had shorter developmental periods (25 and 50 days, respectively) without parboiling, showed extended development times at higher temperatures, particularly 80°C. Yasmin required 52 days, while Super 300 took 33 days.

For milled rice, Sakha 108 and Giza 178, which took 60 days to develop without parboiling, had their developmental times reduced to 47 and 45 days, respectively, at 80°C. In contrast, Super 300 and Yasmin, which initially took 19 and 42 days without parboiling, experienced prolonged developmental periods at higher temperatures, with Super 300 taking 40 days and Yasmin taking 55 days at 80°C.

#### Rice cultivar susceptibility index

Data in Table 6 showed the Super 300 cultivar (1.83) and the Giza 178 cultivar (1.92) had the lowest susceptibility index for unparboiled rice, specifically paddy. Conversely, the Yasmin cultivar (2.79) demonstrated the highest susceptibility index, closely followed by the Sakha 108 cultivar (2.17). Rice’s susceptibility index decreased when exposed to high temperatures, particularly 80 °C. Among the different cultivars, the paddy-parboiled rice of the Super 300 cultivar had the lowest susceptibility index at 75 °C and 80 °C, with a value of 0.00. The susceptibility index of 0.79 for the Giza 178 cultivar and 0.99 for the Sakha 108 cultivar trailed closely behind. The Yasmin cultivar had the highest susceptibility index, at 2.05. Regarding milled rice cultivars, the susceptibility index was lowest for parboiled Sakha 108 and Giza 178 cultivars, with a value of 2.16. The Super 300 cultivar (4.84) and the Yasmin cultivar (3.51) exhibited the highest susceptibility index. When exposed to temperature, rice’s susceptibility index decreased, with the lowest values observed at 80 °C for parboiled rice. Among the several cultivars, the Super 300 cultivar had the lowest susceptibility index (0.55), followed by the Sakha 108 cultivar (1.38) and the Giza 178 cultivar (1.55), while the Yasmin cultivar had the highest susceptibility index (2.29). This study examined rice cultivars Sakha 108, Giza 178, Yasmin (paddy and milled-parboiled; paddy and milled-un parboiled), and Paddy Super 300. We then classified the cultivars as resistant. Table 6 classified the Super 300 milled-un parboiled rice as a moderately resistant cultivar.

## Discussion

The milling process is a crucial stage in rice production, as it significantly impacts the nutritional and cooking qualities of the final product [3, 54]. This study investigates methods to maximize milling recovery percentage economically, with a particular main focus was related to insect infestation. The results indicate that parboiling significantly increases milling recovery, with the optimal recovery achieved at a soaking temperature of 80 °C. Parboiling enhances milling recovery by promoting starch gelatinization and grain expansion, reducing internal cracks, and increasing grain hardness [23, 55]**.** Parboiling has been identified as an important process for improving rice cooking and milling qualities through soaking, steaming, and drying [56]. Odoom [57] stated that there is a very high loss of rice during de-hulling and polishing when using poor-performance machines and unskilled operators. Thus, to solve this problem, parboiling technology has been introduced to harden the kernel and gelatinizes the starch [58]. It was also described that parboiling technology is the hydrothermal processing of paddy rice or brown rice to reduce breakage levels and improve the head rice yield [59]. Gelatinization enhances the removal of the rice husk easily at a low frictional force with minimum breakage [60]. The process of parboiling involves partially boiling rice when it is still in its husk, or when it is in the form of brown rice, before milling to increase its nutritional value, change its appearance, and reduce breakage in milling [61, 62]. This process involves several steps, including soaking, steaming, and drying [62]. It increases the levels of calcium, potassium, iron, and manganese in rice, making it a healthier option compared to white or brown rice. Parboiled rice also contains more vitamins than white or brown rice, with one cup providing the recommended daily intake of these essential nutrients. rice mill’s whitening process. The parboiling process also improves the quality of rice by reducing breakage in milling and enhancing head rice production. This is achieved through the gelatinization of starch during the boiling process, followed by the re-association of amylase molecules during cooling, resulting in a tightly packed structure that makes the kernels harder and glassier [47, 62]. Starch content was found highest in raw rice (82.23 ± 0.23%) as compared to parboiled rice and puffed rice [63]. The reason could be due to the fact that the rice grains are passed through two heating process i.e. parboiling (gelatinization of starch) and puffing at high temperature (270–280 °C). During this process the starch granules are gelatinized and retrograded and some portion of starch is converted into resistant starch [63].

The correlation analysis reveals significant relationships between various milling, cooking, and eating quality traits. The positive correlation between hulling percentage and milling percentage (0.89) underscores the importance of hulling efficiency in determining milling yield. However, the negative correlations between hulling percentage and elongation (− 0.59) and hardness (− 0.85) suggest that higher hulling percentages may be associated with reduced elongation and hardness, which could impact the overall quality of the rice. Milling percentage was negatively correlated with hardness (− 0.83) and other traits, indicating that increased milling yields might lead to reduced grain hardness and other quality parameters. The strong positive correlations between elongation, water uptake, gel consistency, and protein percentage suggest that these traits are interrelated. For instance, elongation showed a significant positive correlation with water uptake (0.98) and gel consistency (0.94), indicating that rice grains with better water uptake tend to elongate more and have higher gel consistency. Similarly, the high correlation between gel consistency and protein percentage (0.95) suggests that rice with better cooking quality also has higher protein content. Our results are in agreement with those of previous studies [64, 65]. Based on the strong correlation between all possible pairs of quality characters, it was concluded that the characters can be employed as selection indicators for the advancement of grain quality traits in rice.

The resistance variability between the parboiled and un-parboiled (paddy and milled) rice of some Egyptian rice cultivars was mainly due to the F_1_ progeny emergence, median developmental time, and susceptibility index. Resistant cultivars exhibited minimum grain-reduced multiplication of F_1_ progeny, a longer median developmental period, and a lower score of the susceptibility index. Parboiling changes the mechanical characteristics of rice grains, contributing to the differences in resistance of rice cultivars to stored grain insect attacks in addition to genetic factors for cultivars. Progeny emergence was highly correlated with the thermal stage of rice parboiling to weevil infestation. Progeny emergence is highly correlated with the thermal conditions of parboiling. Susceptible rice varieties produce a greater number of progeny compared to resistant varieties. Additionally, *Sitophilus oryzae* requires less developmental time on susceptible varieties, whereas resistant varieties have a longer developmental period.Similar findings were reported by [66, 67] used mean developmental time as an indicator of the susceptibility of rice varieties to the attack of *S. oryzae*. The same parameters of adult progeny and developmental period were adopted in rice varieties against *S. oryzae* by Rashid et al. [68]. Based on the mean developmental time and the number of F1 progeny that emerged, this has been used to compute the susceptibility index, and for the categorization of genotypes [69, 70]. In this regard, the results showed that Super 300 milled un-parboiled was classified as moderately resistant, whereas after the thermal stage of parboiling, it was classified as resistant. It can now be concluded that parboiling changes the mechanical characteristics of rice grains, making them less penetrable by weevils [44]. However, conflicting results were observed by Rashid et al. [68] reporting that husked-parboiled rice was favored while un-husked-parboiled rice was least preferred. *S. oryzae* infest ability success was greater in un-husked un-parboiled rice than in husked-parboiled rice.

The study’s findings underscore the practical benefits of integrating parboiling into rice storage and pest management strategies. By optimizing parboiling conditions—particularly at higher temperatures like 75 °C and 80 °C—large-scale rice storage operations can significantly enhance resistance to Sitophilus oryzae, reducing pest-related losses and minimizing the need for chemical treatments. This approach not only helps manage pests more sustainably but also maintains or improves grain quality by optimizing key traits such as milling yield and cooking quality. Selecting resilient rice cultivars and implementing precise parboiling techniques can further enhance these benefits. However, it is essential to balance the costs associated with parboiling, such as energy and equipment, against the benefits of improved pest resistance and grain quality. Educating farmers and storage operators about effective parboiling practices and cultivar selection can ensure the successful adoption of these strategies, contributing to more efficient and sustainable rice storage systems.

## Conclusion

The study highlights the significant influence of parboiling on both the resistance of rice cultivars to *Sitophilus oryzae* and their grain quality characteristics. Parboiling, particularly at higher temperatures, enhances the resistance of certain rice cultivars to rice weevils by reducing progeny emergence and extending developmental times. Cultivars such as Super 300 and Giza 178 demonstrated superior resistance when parboiled at 75 °C and 80 °C, indicating that thermal treatment can effectively enhance grain protection. Conversely, susceptible cultivars like Yasmin displayed higher progeny emergence, reaffirming the variability in pest resistance across different rice varieties. Furthermore, the study establishes significant correlations between milling, cooking, and eating quality traits, suggesting that optimizing parboiling conditions can not only improve resistance to pests but also maintain or enhance the overall quality of rice grains. These findings provide valuable insights for rice breeders and processors in selecting and optimizing rice cultivars and processing methods to achieve both pest resistance and grain quality, contributing to more sustainable rice production systems.

## Data Availability

Data will be made available on request.
